# Total tanshinones exhibits anti-inflammatory effects through blocking TLR4 dimerization via the MyD88 pathway

**DOI:** 10.1038/cddis.2017.389

**Published:** 2017-08-17

**Authors:** Hongwei Gao, Xin Liu, Wen Sun, Naixin Kang, Yanli Liu, Shilin Yang, Qiong-ming Xu, Chunming Wang, Xiuping Chen

**Affiliations:** 1 State Key Laboratory of Quality Research in Chinese Medicine, Institute of Chinese Medical Sciences, University of Macau, Macau, China; 2 Department of Pharmacognosy, College of Pharmaceutical Science, Soochow University, Suzhou 215123, China

## Abstract

Tanshinones belong to a group of lipophilic constituents of *Salvia miltiorrhiza* Bunge (Danshen), which is widely used in traditional Chinese medicine. A deluge of studies demonstrated that tanshinones exert anti-inflammatory effects, but the underlying mechanisms remain unclear to date. This study investigated the anti-inflammatory effects and mechanisms of total tanshinones (TTN). TTN suppressed the expression of cyclooxygenase-2 (COX-2) and inducible nitric oxide synthase (iNOS) and the secretion of TNF-*α*, IL-6, and IL-1*β* in RAW264.7 cells, bone marrow-derived macrophages, and THP-1 cells. TTN attenuated the LPS-induced transcriptional activity of NF-*κ*B and decreased I*κ*B-*α* and IKK phosphorylation and NF-*κ*B/p65 nuclear translocation. Furthermore, TTN inhibited the LPS-induced transcriptional activity of AP-1, which was induced by the reduction of JNK1/2, ERK1/2, and p38MAPK phosphorylation. TTN blocked LPS-induced Toll-like receptor 4 (TLR4) dimerization, which consequently decreased MyD88 recruitment and TAK1 phosphorylation. In addition, TTN pretreatment effectively inhibited xylene-induced ear edema and LPS-induced septic death and improved LPS-induced acute kidney injury in mice. TTN exerts anti-inflammatory effects *in vitro* and *in vivo* by blocking TLR4 dimerization to activate MyD88–TAK1–NF-*κ*B/MAPK signaling cascades, which provide the molecular basis of the anti-inflammatory effect of Danshen and suggest that TTN is a potential agent for the treatment of inflammatory diseases.

Macrophages, the immune cells against invading pathogens, have a cardinal role in innate immune response.^1, 2^ However, excessively activated macrophages usually cause the aberrant release of inflammatory mediators involved in diversified inflammatory diseases, such as rheumatoid arthritis, sepsis, and inflammatory bowel disease.^3, 4, 5^ Thus the inhibition of inflammatory mediators such as TNF-*α*, inducible nitric oxide synthase (iNOS), cyclooxygenase-2 (COX-2), IL-1*β*, and IL-6 is the priority in the development of new anti-inflammatory drugs. The transcriptional factor NF-*κ*B contributes to the initiation and amplification of inflammation.^6, 7^ NF-*κ*B proteins in the cytoplasm are inactive because they bind to inhibitory proteins I*κ*Bs. Immediately after NF-*κ*B is activated, the active form composed of p65 and p50 subunits translocates to the nucleus to instigate the release of pro-inflammatory mediators.^8, 9^ In addition, another transcriptional factor, AP-1, is mediated by MAPK families such as p38MAPK, JNK, and ERK in response to cellular inflammatory stimuli that regulate pro-inflammatory mediators.^7, 10, 11^ Thus the suppression of the NF-*κ*B or MAPK pathway, which blocks the release of inflammatory cytokines, is an important strategy to develop new anti-inflammatory drugs.

Recent studies have shown that the NF-*κ*B and MAPK pathways are mediated by Toll-like receptor 4 (TLR4) dimerization in the cellular membrane.^12, 13^ TLR4, as a critical signaling receptor for LPS, has a vital role in mediating innate and acquired immunity.^14^ Activated by LPS, TLR4 can form a dimer to recruit MyD88 and/or TRIF.^15^ With regard to the MyD88-dependent pathway, activated TAK1 subsequently leads to translocate, synthesize, and release pro-inflammatory mediators through the NF-*κ*B and/or MAPK pathways.^16^ Therefore, the inhibition of TLR4 homodimerization is proposed to be an alternative strategy to treat inflammatory disorders.

Sepsis and subsequent multiple organ dysfunction, the leading cause of death among patients, are characterized by whole-body inflammatory response.^17, 18^ Acute kidney injury (AKI), a devastating result of sepsis,^19^ is a worldwide heath problem with no effective drug available for its treatment. LPS is an important factor that leads to AKI.^20^ LPS can upregulate the production of pro-inflammatory cytokines such as TNF-*α*, IL-6, and IL-1*β*, which promote the development of AKI.^21, 22^

Total tanshinones (TTN) is a group of lipid-soluble ingredients isolated from the roots of *Salvia miltiorrhiza* Bunge (Danshen). Danshen is widely used in traditional Chinese medicine for the treatment of cardiovascular and inflammatory diseases.^23^ Many drugs containing Danshen such as Tanshinone capsule and Fu Fang Danshen dripping pill have been approved by the China Food and Drug Administration. Of note, Fu Fang Danshen dripping pill has been approved for phase 3 clinical trials by the FDA in the United States. Tanshinone IIA and cryptotanshinone, two main TTN constituents, have been corroborated to show anti-inflammatory effects.^23, 24^ However, their anti-inflammatory effect and mechanisms have not been illustrated. Therefore, the present study investigated the anti-inflammatory effect and mechanisms of TTN *in vitro* and *in vivo*.

## Results

### TTN suppressed LPS-induced iNOS and COX-2 expression in RAW264.7 cells

**Figure 1:**
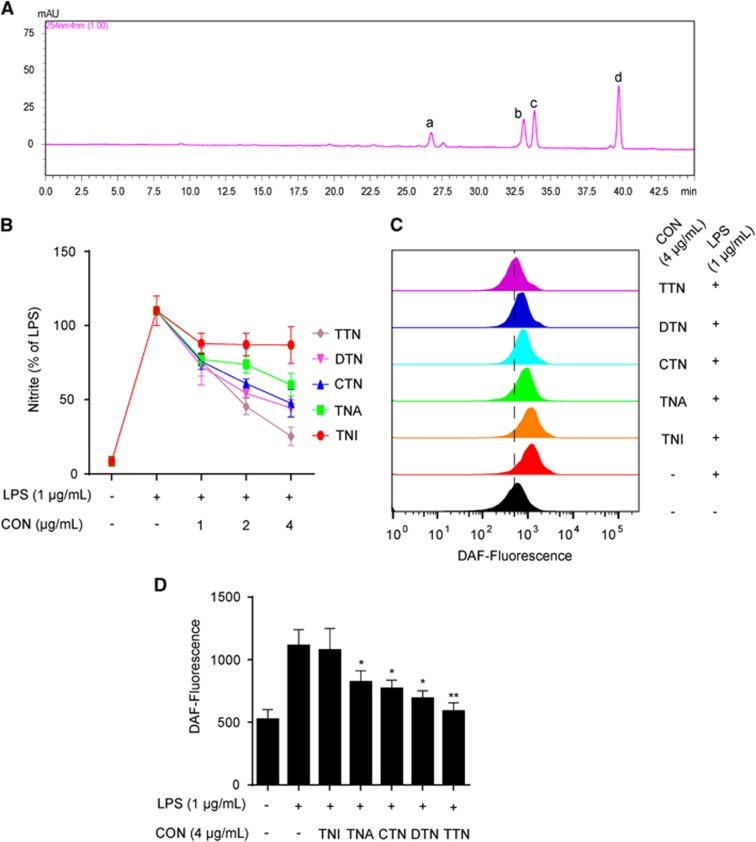
TTN exhibited anti-inflammatory effects in lipopolysaccharide (LPS)-stimulated RAW264.7 cells. (**a**) HPLC chromatogram of TTN. (a) DTN (5.34%); (b) TNI (19.81%); (c) CTN (22.12%); (d) TNA (45.12%). (**b**) RAW264.7 cells were pretreated with TNI, TNA, CTN, DTN, or TTN for 1 h before LPS (1 *μ*g/ml) stimulation for another 24 h. Nitrite production was determined by Griess assay. (**c** and **d**) RAW264.7 cells were pretreated with TNI (4 *μ*g/ml), TNA (4 *μ*g/ml), CTN (4 *μ*g/ml), DTN (4 *μ*g/ml), or TTN (4 *μ*g/ml) for 1 h before LPS (1 *μ*g/ml) stimulation for another 6 h. NO was determined by flow cytometry with DAF-FM (1 *μ*M). The values were expressed as means±S.D. *P<0.05 and **P<0.01 *versus* LPS alone group, *n*=6

**Figure 2:**
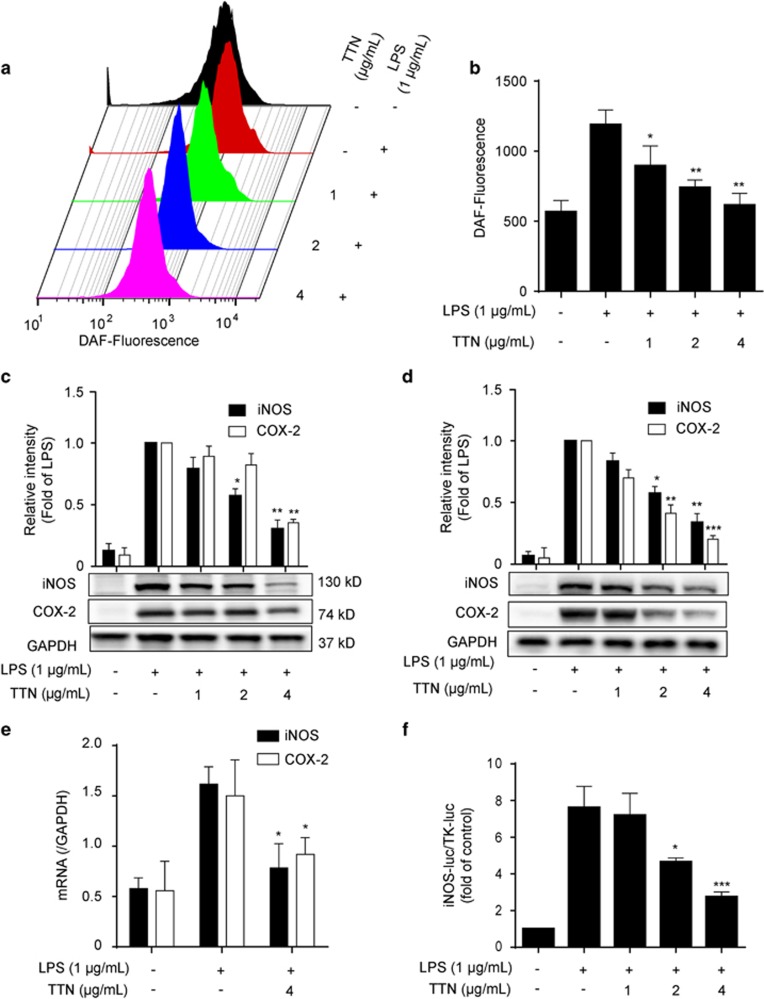
TTN suppressed lipopolysaccharide (LPS)-induced iNOS and COX-2 expression in RAW264.7 and THP-1 cells. (**a** and **b**) RAW264.7 cells were pretreated with TTN for 1 h before LPS (1 *μ*g/ml) stimulation for another 6 h. NO was determined by flow cytometry with DAF-FM (1 *μ*M) (*n*=6). (**c** and **d**) RAW264.7 and THP-1 cells pretreated with the indicated concentrations of TTN for 1 h before LPS (1 *μ*g/ml) stimulation for another 24 h. The expression levels of iNOS and COX-2 were examined by western blotting analysis (*n*=6). (**e**) RAW264.7 cells were pretreated with TTN (1–4 *μ*g/ml) for 1 h before LPS stimulation for another 6 h. The mRNA levels of iNOS and COX-2 were determined by qRT-PCR assay (*n*=6). (**f**) RAW264.7 cells were transiently co-transfected with iNOS-luc and TK-luc for 48 h. Cells were pretreated with TTN (4 *μ*g/ml) before LPS (1 *μ*g/ml) stimulation for another 24 h. Luciferase activity was determined by Dual-Glo Luciferase Assay (*n*=6). The values were expressed as means±S.D. *P<0.05, **P<0.01, and ***P<0.001 *versus* LPS alone group

As shown in Figure 1A, four peaks in the HPLC chromatogram were validated as dihydrotanshinone (DTN, 5.34%), tanshinone I (TNI, 19.81%), cryptotanshinone (CTN, 22.12%), and tanshinone IIA (TNA, 45.12%). The anti-inflammatory activities of these pure compounds and TTN were investigated using LPS-stimulated RAW264.7 cells. As shown in Figure 1B, TTN and DTN could significantly decrease LPS-induced nitrite levels, whereas the effects of TNI, TNA, and CTN were much weaker. Similar to nitrite level, TTN displayed more significant inhibitory effect on nitric oxide (NO) level than the pure compounds (Figures 1C and D). Thus TTN was chosen for further study. MTT assay showed that all the tested compounds have no significant cytotoxic effect on RAW264.7 cells after 24 h treatment (Supplementary Figure S1). TTN suppressed LPS-induced NO production in a concentration-dependent manner (Figures 2a and b). The LPS-stimulated protein expression of iNOS and COX-2 in RAW264.7 and THP-1 cells was also inhibited by TTN pretreatment (Figures 2c and d). Furthermore, TTN significantly inhibited LPS-induced iNOS and COX-2 mRNA expression (Figure 2e). In addition, TTN suppressed LPS-induced iNOS promoter activity in a concentration-dependent manner (Figure 2f).

### TTN inhibited the release of LPS-induced cytokines

**Figure 3:**
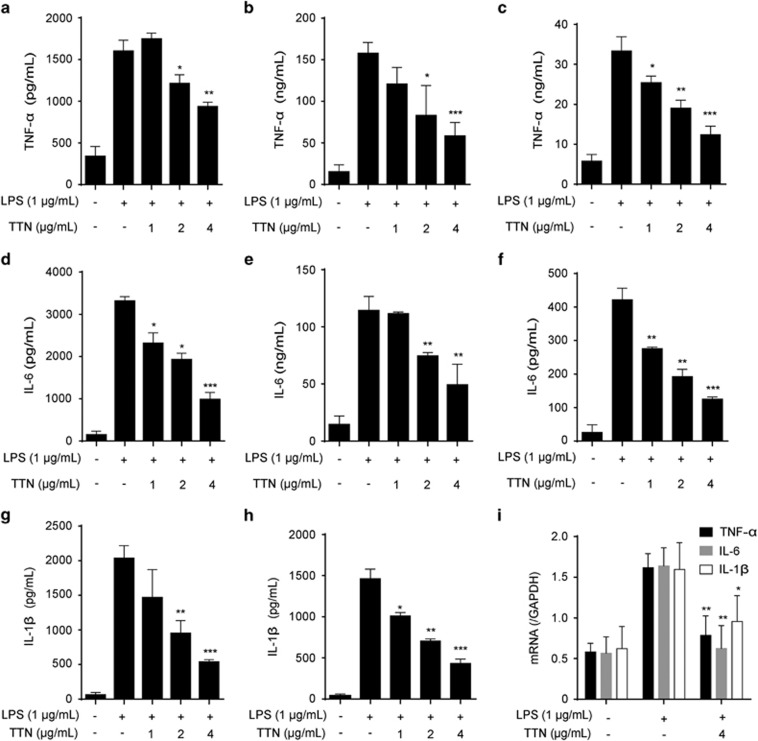
TTN suppressed the release of lipopolysaccharide (LPS)-induced pro-inflammatory cytokines in RAW264.7 cells, BMDMs, and THP-1 cells. (**a** and **d**) RAW264.7 cells were pretreated with the indicated concentrations of TTN for 1 h before LPS stimulation for another 24 h. Tumor necrosis factor (TNF)-*α* and interleukin (IL)-6 were determined by ELISA assay (*n*=6). (**b**, **e**, **g**) BMDMs were pretreated with the indicated concentrations of TTN for 1 h before LPS stimulation for another 24 h. TNF-*α*, IL-6, and IL-1*β* were determined by ELISA assay (*n*=6). (**c**, **f**, **h**) THP-1 cells were pretreated with the indicated concentrations of TTN for 1 h before LPS stimulation for another 24 h. TNF-*α*, IL-6, and IL-1*β* were determined by ELISA assay (*n*=6). (**i**) RAW264.7 cells were pretreated with TTN for 1 h before LPS stimulation for another 6 h. mRNA levels of TNF-*α*, IL-6, and IL-1*β* were detected by qRT-PCR assay. The values were expressed as means±S.D. *P<0.05, **P<0.01, and ***P<0.001 *versus* LPS alone group

LPS-stimulated production of pro-inflammatory mediators such as NO, TNF-*α*, IL-6, and IL-1*β* via the NF-*κ*B and/or MAPK pathway is easily detected in macrophage cells.^25, 26^ TTN pretreatment significantly decreased the levels of TNF-*α* (Figures 3a–c), IL-6 (Figures 3d–f), and IL-1*β* (Figures 3g and h) in culture medium of RAW264.7 cells, bone marrow-derived macrophages (BMDMs), and THP-1 cells in a concentration-dependent manner. Furthermore, their mRNA expression was significantly inhibited by TTN (Figure 3i).

### TTN inhibited LPS-induced NF-*κ*B and AP-1 activation

**Figure 4:**
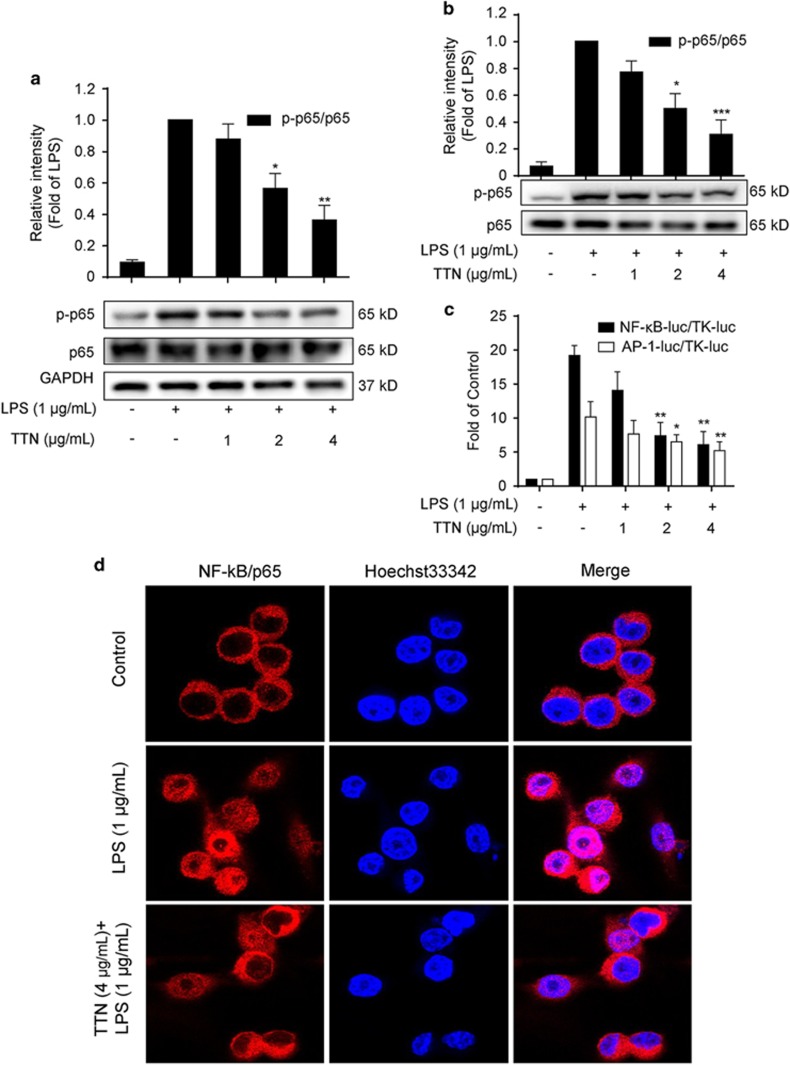
Effects of TTN on lipopolysaccharide (LPS)-induced NF-*κ*B and AP-1 activation (**a** and **b**) RAW264.7 cells and THP-1 cells were pretreated with TTN for 1 h before LPS stimulation for another 2 h. The phosphorylation of p65 was determined by western blotting analysis (*n*=6). (**c**) RAW264.7 cells were transiently co-transfected with NF-*κ*B-luc and TK-luc or AP-1-luc and TK-luc for 48 h. Cells were pretreated with TTN (4 *μ*g/ml) before LPS (1 *μ*g/ml) stimulation for another 24 h. Luciferase activity was determined by Dual-Glo Luciferase Assay (*n*=6). (**d**) RAW264.7 cells were pretreated with the indicated concentrations of TTN for 1 h before LPS stimulation for another 2 h. NF-*κ*B/p65 translocation was determined by immunofluorescence assay (*n*=3). The values were expressed as means±S.D. *P<0.05 and **P<0.01 *versus* LPS alone group

NF-*κ*B and AP-1 activation has pivotal roles in inflammation.^8^ As shown in Figures 4a and b, LPS significantly induced NF-*κ*B p65 phosphorylation, which was inhibited by TTN in RAW264.7 and THP-1 cells. Furthermore, reporter gene assay showed that TTN significantly suppressed NF-*κ*B and AP-1 luciferase activities in a concentration-dependent manner (Figure 4c). In addition, TTN inhibited the LPS-induced translocation of NF-*κ*B p65 into the nucleus as shown by immunofluorescence staining analysis (Figure 4d).

### TTN induced I*κ*B-*α* degradation and IKK-*α*/*β* activation in RAW264.7 cells

**Figure 5:**
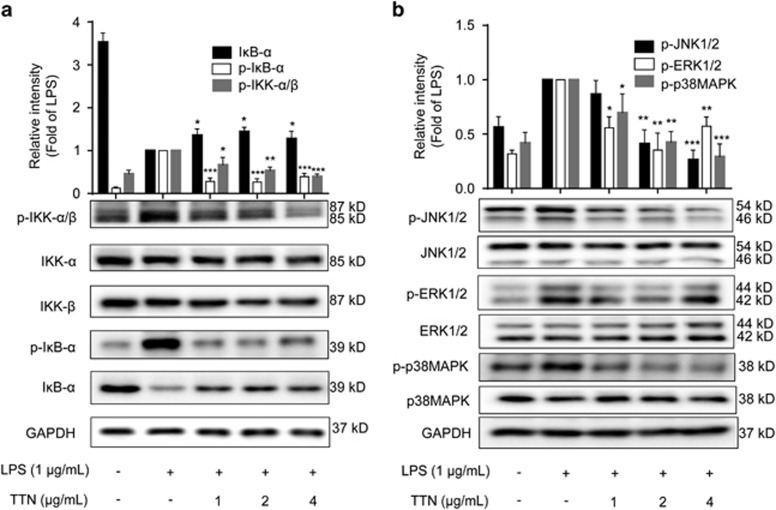
Effects of TTN on the lipopolysaccharide (LPS)-induced degradation of I*κ*B-*α* and activation of the IKK-*α*/*β* and MAPK pathways. (**a** and **b**) RAW264.7 cells were pretreated with TTN for 1 h before LPS (1 *μ*g/ml) stimulation for another 2 h. The expression levels of I*κ*B-*α*, IKK-*α*/*β*, IKK-*α*, IKK-*β*, p-JNK1/2, JNK1/2, p-ERK1/2, ERK1/2, p-p38MAPK, and p38MAPK were determined by western blotting analysis (*n*=5). The values were expressed as means±S.D. *P<0.05, **P<0.01, and ***P<0.001 *versus* LPS alone group

I*κ*B-*α*, the inhibitor protein of NF-*κ*B, makes it stay in inactive state in the cytoplasm. However, after exposure to stimuli such as LPS, I*κ*B-*α* becomes phosphorylated at specific sites resulting in polyubiquitination and proteasomal degradation, which allows the free NF-*κ*B to translocate from the cytoplasm to the nucleus.^27, 28^ As shown in Figure 5a, LPS induced the phosphorylation and degradation of I*κ*B-*α*, which were significantly inhibited by TTN pretreatment. Furthermore, the LPS-induced phosphorylation of IKK-*α* and IKK-*β*, two upstream kinases of I*κ*B in the NF-*κ*B signaling pathway, was sharply decreased by TTN without affecting the total IKK-*α*/*β*.

### TTN inhibited LPS-induced MAPK phosphorylation in RAW264.7 cells

The activation of MAPK (JNK1/2, ERK1/2, and p38MAPK) signaling pathways is always a response to inflammatory stress.^29^ Furthermore, the phosphorylation of MAPKs activates c-Jun, leading to its translocation into the nucleus and its binding to Jun or Fos family members to form AP-1 transcriptional factor.^30^ As shown in Figure 5b, LPS dramatically induced the expression of p-JNK1/2, p-ERK1/2, and p-p38MAPK, which was significantly suppressed by TTN. TTN showed no effect on the total expression of JNK1/2, ERK1/2, and p38MAPK.

### TTN disrupted LPS-induced TLR4 dimerization in RAW264.7 cells

**Figure 6:**
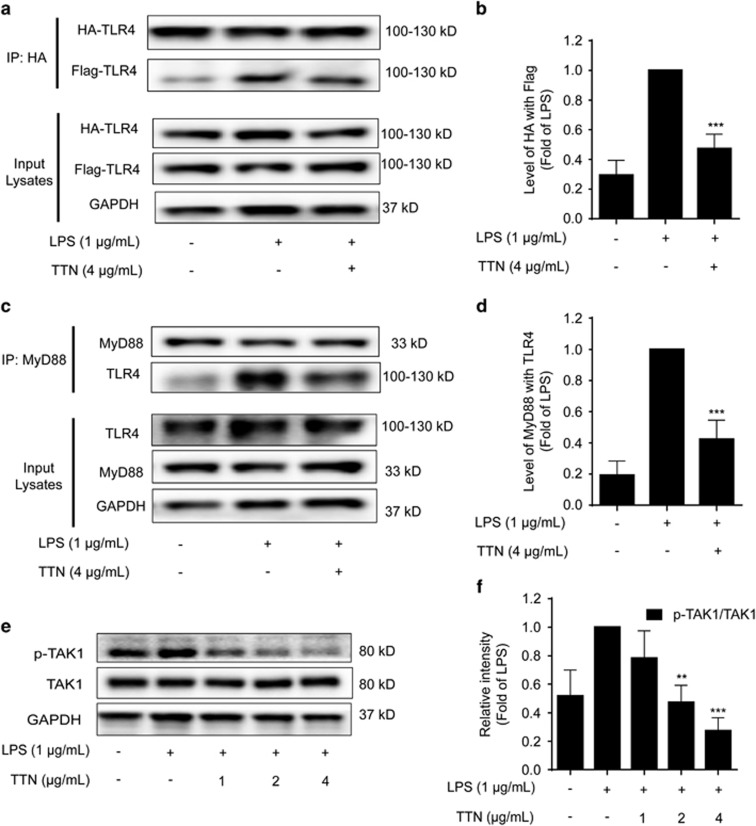
TTN blocked lipopolysaccharide (LPS)-induced TLR4 dimerization involving the MyD88 pathway. (**a**) HEK293T cells were co-transfected with TLR4-HA and TLR4-Flag for 24 h. Cells were pretreated with TTN (4 *μ*g/ml) for 1 h before LPS (1 *μ*g/ml) stimulation for another 24 h. The extent of TLR4 dimerization was determined by co-immunoprecipitation assay. Collected proteins were immunoprecipitated with Anti-HA Magnetic Beads. Immunocomplexes were determined by western blotting analysis with anti-HA and anti-Flag antibodies (*n*=5). (**b**) Quantification of TLR4-HA and TLR4-Flag in HEK293T cells. Quantification of TLR4-HA and TLR4-Flag was detected by densitometric analysis, and TLR4-Flag was compared with TLR4-HA. (**c**) RAW264.7 cells were pretreated with TTN (4 *μ*g/ml) for 1 h before LPS (1 *μ*g/ml) stimulation for another 2 h. Collected proteins were immunoprecipitated with MyD88 using magnetic beads. Immunocomplexes were determined by western blotting analysis with anti-MyD88 and anti-TLR4 antibodies (*n*=5). (**d**) Quantification of MyD88 and TLR4 was detected by densitometric analysis, and TLR4 was compared with MyD88. (**e** and **f**) RAW264.7 cells were pretreated with TTN (4 *μ*g/ml) for 1 h before LPS (1 *μ*g/ml) stimulation for another 2 h. The expression of p-TAK1 and TAK1 was examined by western blotting analysis. The values were expressed as means±S.D. **P<0.01 and ***P<0.001 *versus* LPS alone group

TLR4, a transmembrane receptor expressed on the surface of immune cells, has a pivotal role in regulating innate and acquired immunity and inflammation.^31^ Stimulated by LPS, TLR4 forms a dimer and then activates the NF-*κ*B and/or MAPK pathway, leading to a pathogen-specific innate immune response via the release of pro-inflammatory cytokines.^32^ To determine whether LPS-induced TLR4 dimerization could be affected by TTN, HEK293T cells were co-transfected with TLR4-HA and TLR4-Flag plasmids. As shown in Figures 6a and b, compared with the LPS-treated group, the decrease of TLR4-Flag in TLR4-HA precipitation after TTN pretreatment suggested that TTN blocked LPS-induced TLR4 dimerization.

### TTN blocked LPS-induced MyD88-dependent signaling cascades in RAW264.7 cells

TLR4 dimerization triggers two pathways during the pro-inflammatory process: the MyD88-dependent and MyD88-independent pathways.^32^ The MyD88-dependent pathway is initiated from the recruitment of MyD88 to the Toll/interleukin receptor domain of TLR/IL-1R and then binds with IRAK4, enabling IRAK1 to recruit TRAF6. The IRAK1–TRAF6 complex phosphorylates TAB2/TAB3 and TAK1 and thus leads to the activation of the NF-*κ*B and MAPK signaling pathway.^33^ We explored whether TTN exerted effects on the LPS-induced MyD88 pathway. As shown in Figures 6c and d, LPS significantly increased the formation of the TLR4–MyD88 complex, which could be decreased by TTN. In addition, TTN inhibited the LPS-induced phosphorylation of TAK1 without affecting the total TAK1 (Figures 6e and f).

### TTN inhibited inflammation *in vivo*

**Figure 7:**
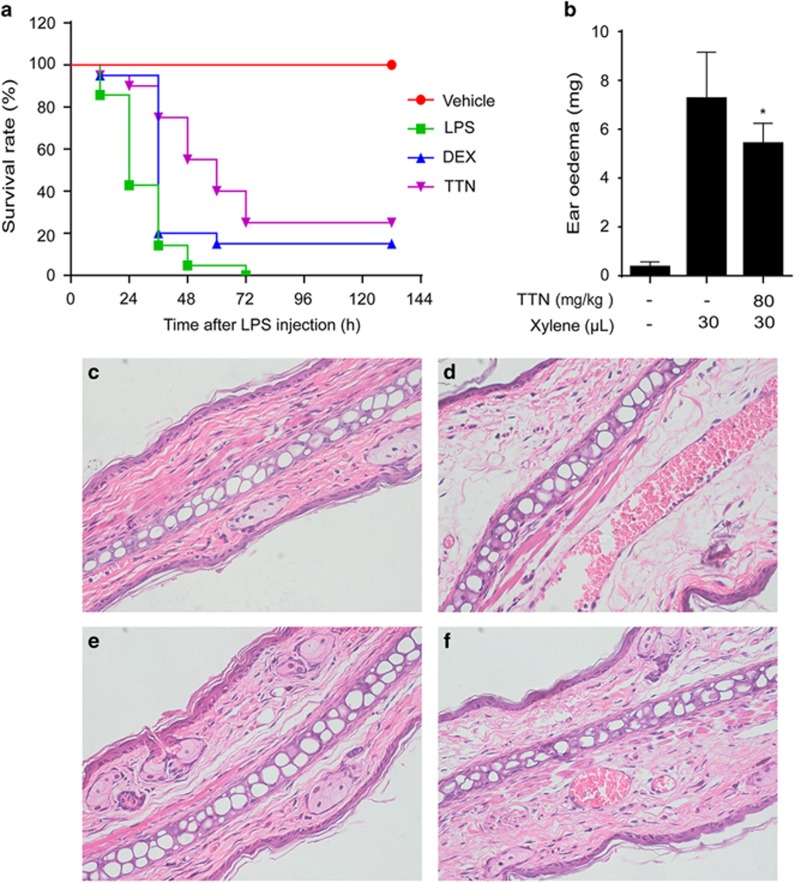
TTN decreased xylene-induced ear edema and reduced lipopolysaccharide (LPS)-induced septic shock in mice. (**a**) Ear edema was established by xylene administration. Pretreatment with TTN (80 mg/kg) for 2 h before xylene injection. One hour later, ear weight was measured (*n*=10). Values were expressed as means±S.D. *P<0.05 *versus* xylene group. (**b**) Twenty mice per group pretreated with vehicle or TTN (80 mg/kg, intraperitoneal (i.p.)) for 2 h before LPS (20 mg/kg, i.p.) injection. DEX, positive. Survival rates of these mice were observed for the next 132 h. DEX (*n*=20). The ear tissues were detected by H&E staining (magnification, × 400). (**c**) The left ear of mice without TTN treatment was regarded as a vehicle control. (**d**) The right ear of mice treated with xylene (30 *μ*l) and without TTN was regarded as the model control. (**e**) The left ear of mice pretreated with TTN (80 mg·kg^−1^, i.p.) for 2 h was not applied with xylene. (**f**) The right ear of mice pretreated with TTN (80 mg·kg^−1^, i.p.) for 2 h was treated with xylene (30 *μ*l) for another 1 h

**Figure 8:**
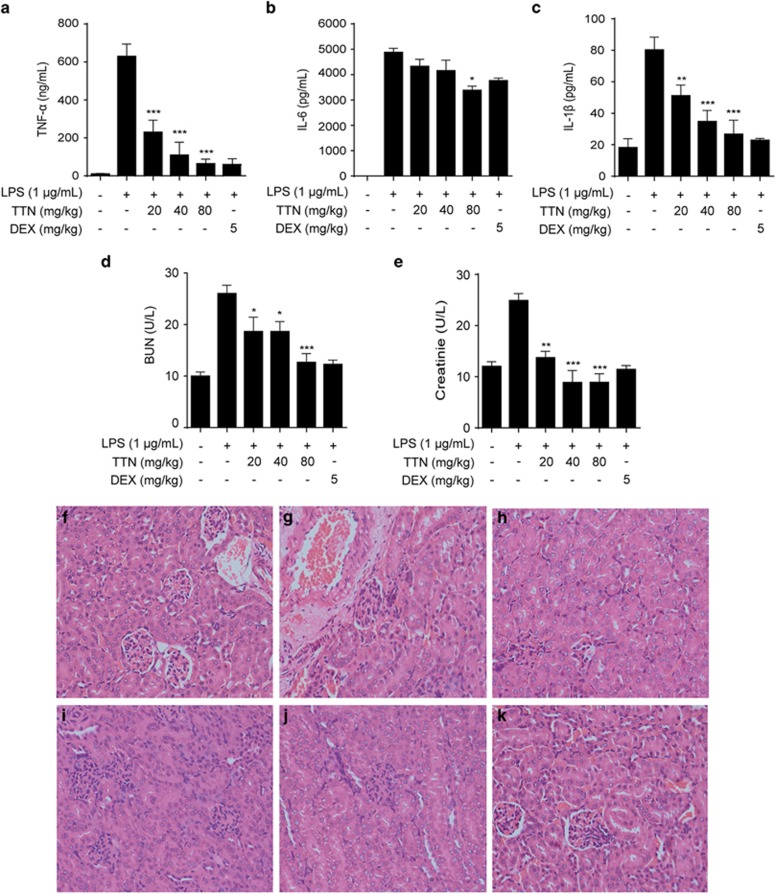
TTN suppressed the release of inflammatory cytokines and renal injury and improved kidney injury in lipopolysaccharide (LPS)-stimulated AKI mice. BALB/c mice were pretreated with TTN (20, 40, and 80 mg/kg, intraperitoneal (i.p.)) for 2 h before LPS (10 mg/kg, i.p.) injection. After 12 h, blood samples were collected via the retro-orbital route under anesthesia. Tumor necrosis factor-*α* (**a**), interleukin (IL)-6 (**b**), and IL-1*β* (**c**) were determined by ELISA kits, and blood urea nitrogen (**d**) and creatinine (**e**) were examined by Roche Modular P800. H&E staining (magnification, × 400). (**f**) Control group; (**g**) LPS (10 mg/kg, i.p.) group; (**h**) TTN (20 mg/kg, i.p.) group; (**i**) TTN (40 mg/kg, i.p.) group; (**j**) TTN (80 mg/kg, i.p.) group; (**k**) DEX (5 mg/kg, i.p.). DEX, positive control (*n*=10). The values were expressed as means±S.D. *P<0.05, **P<0.01, and ***P<0.001 *versus* LPS alone group

In the xylene-induced ear edema mouse model, the significant inhibitory effect of TTN was observed on ear weight (Figure 7a) and hematoxylin and eosin (H&E)-stained ear sections (Figures 7c–f). Furthermore, in the LPS-induced sepsis model, all mice died within 72 h, whereas TTN pretreatment could significantly increase the survival rate to 25%, which was more effective than dexamethasone (DEX) (Figure 7b). In addition, TTN significantly reduced the serum levels of cytokines TNF-*α*, IL-6, and IL-1*β* in the LPS-induced AKI model (Figures 8a–c). The serum levels of creatinine and blood urea nitrogen (BUN) were also significantly suppressed by TTN (Figures 8d and e). Compared with the control group (Figure 8f), LPS induced edema of renal tubular epithelial cells and glomerular atrophy, the dilation of renal capsule cavity, and the destruction of tubular structures. The epithelial cells of local focal necrosis collapse and renal interstitial edema of epithelial cells were observed (Figure 8g). These pathological alterations were improved by TTN (Figures 8h–j) and DEX pretreatment (Figure 8k).

## Discussion

For decades, the lipophilic tanshinones were proposed to be the active components of Danshen. Moreover, the anti-inflammatory activities of four major tanshinones (TNI, TNA, CTN, and DTN) have been reported.^23, 24^ However, owing to similarities in their structures, purifying them is expensive and time consuming. Thus we first prepared TTN and compared its anti-inflammatory activity with its compositions. Interestingly, TTN exhibited better efficacy than all of its single compound in the LPS-stimulated RAW264.7 cell model. This finding indicated the existence of enhanced and/or synergetic effects among these compounds. Thus TTN was selected for further investigation. Our results showed that TTN exhibits anti-inflammatory effects *in vitro* and *in vivo* through blocking TLR4 dimerization via the MyD88 pathway.

TLR4, one of the first TLR family members identified in 1997,^34^ was considered as the gene encoding the LPS receptor.^35, 36^ TLR4 in association with MD-2 is responsible for the physiological recognition of LPS in many different cells that express TLR4 and MD-2, such as macrophages; lymphoid cells; and epithelial, endothelial, and vascular smooth muscle cells.^31^ Two copies of the TLR4–MD-2–LPS complex displayed a symmetrical manner to activate the pro-inflammatory signaling pathways.^37^ Therefore, inhibiting TLR4 dimerization is proposed to be a new strategy in treating inflammatory disorders. Many previous studies indicated that LPS could increase the expression of TLR4 and some compounds could reverse the effect of LPS on TLR4 in RAW264.7 cells, suggesting a new anti-inflammatory mechanism.^31^ Nevertheless, we believe that blocking the dimerization of TLR4 is an alternative strategy for anti-inflammation. In this study, TTN could significantly block the dimerization of TLR4 using two different plasmids, TLR4-HA and TLR4-Flag (Figures 6a and b).

Dimerized TLR4 recruits two adaptor protein pairs: MAL–MyD88 and TRAM–TRIF. MAL–MyD88 is needed to activate the NF-*κ*B and MAPK pathways and produce pro-inflammatory cytokines, such as TNF-*α*, IL-1*β*, and IL-6.^31^ The present data indicated that TTN could decrease the LPS-induced interaction between TLR4 and MyD88 in RAW264.7 cells (Figures 6c and d).

The activation of transcription factors NF-*κ*B and AP-1 in the TLR4–MyD88 pathway involves TAK1 and two adaptor proteins TAB1 and TAB2.^32^ TAK1, one of the mitogen-activated protein kinase kinase kinase family members, is proven indispensable for NF-*κ*B and AP-1 activation.^32^ In the present study, TTN suppressed the LPS-induced phosphorylation of TAK1 without affecting the total TAK1 in RAW264.7 cells (Figure 6e), which indicated that TAK1 is downstream of TLR4–MyD88 in LPS-stimulated RAW264.7 cells treatment with TTN.

The IKK complex is phosphorylated by active TAK1 released from the TAK1–TAB1/2 complex from the membrane, which leads to the phosphorylation of I*κ*B for degradation.^38^ Our data indicated that TTN attenuated the LPS-induced phosphorylation of IKK-*α*/*β* and I*κ*B-*α* in RAW264.7 cells (Figure 5a). Additionally, TTN decreased LPS-induced I*κ*B-*α* degradation (Figure 5a). The decrease in LPS-induced phosphorylation of NF-*κ*B/p65 led to the suppression of p65 translocation from the cytosol to the nucleus (Figures 4a). In addition, the reporter gene assay indicated that TTN significantly inhibited LPS-induced p65-luc activity in RAW264.7 cells (Figure 4c). A deluge of reports demonstrated that MAPKs are also activated by TAK1 phosphorylation.^38^ Previous studies showed that some novel agents are effective in treating inflammatory diseases through the JNK and p38MAPK signaling pathways.^39^ AP-1 activity is modulated by different MAPK members. As reported, JNK phosphorylation activates the transcriptional potential of c-Jun, a critical part of AP-1.^40^ We noted that JNK1/2, ERK1/2, and p38MAPK contribute to LPS-induced inflammation. TTN inhibited the LPS-induced phosphorylation of JNK, ERK, and p38MAPK (Figure 5b), which accorded with the suppression of AP-1 activity by TTN in LPS-stimulated RAW264.7 macrophages using reporter gene assay (Figure 4c).

To evaluate the effect of TTN on NF-*κ*B- and/or MAPK-regulated gene transcription, we demonstrated that TTN suppressed the LPS-induced expression of inflammatory mediators, such as iNOS, COX-2, TNF-*α*, IL-6, and IL-1*β*. TTN could suppress the induction of iNOS expression through the downregulation of their promoter activities and subsequent production of NO. In addition, TTN inhibits LPS-induced COX-2 expression levels at the transcription and protein levels in RAW264.7 cells. Our findings also indicate that TTN inhibits the expression levels of TNF-*α*, IL-6, and IL-1*β* at the transcription level in a concentration-dependent manner, with the associated reduction of TNF-*α*, IL-6, and IL-1*β* in RAW264.7 cells, DMDMs, and THP-1 cells. These results indicated that the suppression of NF-*κ*B activation by TTN might inhibit pro-inflammatory gene expression at the transcriptional level.

To further confirm the anti-inflammatory effect of TTN, three types of inflammation mouse models were performed. Activation of immunity induced by xylene is dependent on TLR4, where prolonged inflammatory responsiveness can induce systemic inflammatory syndromes, such as edema and sepsis.^41^ In the present study, TTN suppressed xylene-induced ear edema (Figures 7a, c–f), indicating that TTN could attenuate acute inflammation by inhibiting the infiltration of inflammatory cells and the production of pro-inflammatory mediators. To examine the effect of TTN on systemic inflammatory response, the protective effect of TTN on LPS-induced sepsis model was evaluated in our laboratory. Results showed that TTN can significantly rescue mice, and its effect was better than that of DEX used in the clinical treatment of inflammation (Figure 7b). Pretreatment with TTN could alleviate the release of cytokines such as IL-1*β*, IL-6, and TNF-*α* in a dose-dependent manner in serum and improve survival rate (Figures 8a–c). Additionally, the septic AKI caused by aberrant inflammatory response usually leads to death.^21^ In the present study, pretreatment with TTN could attenuate LPS-induced BUN and serum creatinine (Figures 8d and e). H&E staining indicated that TTN could ameliorate AKI induced by LPS in a dose-dependent manner (Figures 8f–k).

In summary, our data indicated that TTN displays anti-inflammatory effects *in vitro* and *in vivo*. The underlying mechanisms might be related to the NF-*κ*B and MAPK pathway via blocking TLR4 dimerization involved in MyD88 signal cascades. TTN is a potential agent for inflammatory diseases.

## Materials and methods

### Materials

TNI (>98%), TNA (>98%), CTN (>98%), and DTN (>98%) purchased from Shun Bo Biological Engineering Technology Co., Ltd. (Shanghai, China) were determined by HPLC. LPS (*Escherichia coli*, serotype 0111:B4), Griess reagent, phorbol 12-myristate 13-acetate (PMA), and antibodies against HA and Flag were purchased from Sigma-Aldrich (St. Louis, MO, USA). Dulbecco’s modified Eagle’s medium (DMEM) and fetal bovine serum (FBS) were purchased from Life Technologies/Gibco Laboratories (Grand Island, NY, USA). ELISA kits for IL-6, IL-1*β*, and TNF-*α* were purchased from Neobioscience (Shenzhen, China). Antibodies against iNOS, COX-2, p65, p-p65, p-IKK-*α*/*β*, IKK-*α*, IKK-*β*, p-I*κ*B-*α*, I*κ*B-*α*, p-p38MAPK, p38MAPK, p-ERK1/2, ERK1/2, p-JNK1/2, JNK1/2, p-TAK1, TAK1, and GAPDH were purchased from Cell Signaling Technology (Beverly, MA, USA). Antibodies against TLR4 and MyD88 were purchased from Santa Cruz Biotechnology (Santa Cruz, CA, USA). Oligonucleotide primers for iNOS, COX-2, TNF-*α*, IL-6, IL-1*β*, and GAPDH were purchased from KeyGEN Biotech (Jiangsu, China). 4-Amino-5-methylamino-2′,7′-dichlorofluorescein diacetate (DAF-FM), Magnetic HA-Tag IP/Co-IP Kit, and Protein A/G Magnetic Beads were purchased from Life Technologies/Thermo Fisher Scientific (Grand Island, NY, USA). NF-*κ*B-luc, iNOS-luc, AP-1-luc, and TK were purchased from Addgene (Beijing, China). Reverse transcriptase PCR, quantitative PCR, and Lipofectamine LTX Kits were purchased from Promega (Madison, WI, USA).

### Preparation of TTN

Dried Danshen (100 kg) was extracted twice with 95% EtOH under reflux. The CH_2_Cl_2_ extract (5.2 kg) was further vacuum chromatographed on a silica gel (60–100 mesh) column (120 × 20 cm^2^ i.d.) and then eluted with petroleum ether/EtOAc (9:1) to obtain TTN. TTN components were identified by HPLC with a UV2401 spectrometer (Shimadzu Corp., Kyoto, Japan). The mobile phase consisted of methanol (A) and water (B, with 0.1% acetic acid) with a flow of 1 ml/min using the subsequent gradient elution: 0–45 min, 55–90% A. The injection volume was 20 *μ*l, and the absorbance wavelength was selected at 254 nm.

### Cell culture

RAW264.7 macrophages were purchased from the Cell Bank of the Chinese Academy of Sciences (Shanghai, China). THP-1 and HEK293T cells were purchased from American Type Culture Collection (Manassas, VA, USA). RAW264.7 and HEK293T cells were cultured in DMEM with 10% FBS. THP-1 cells were cultured in RPIM-1640 with 10% FBS and 50 *μ*M *β*-mercaptoethanol. In experiments, the THP-1 cells were incubated with PMA (100 ng/ml) overnight. All cells were maintained at 37 °C under a humidified atmosphere of 5% CO_2_ in an incubator.

### Isolation of BMDMs

BMDMs from 6-to-9-week-old female BALB/c mice were produced as reported previously.^42^ In brief, mouse bone marrow cells were differentiated in DMEM supplemented with 10% FBS and 20% M-CSF-conditioned medium from L929 cells for 7 days. The BMDMs were then cultured overnight in 6- and 96-well plates at densities of 8 × 10^5^ and 2 × 10^4^ cells/well, respectively.

### MTT assay

RAW264.7 cells were seeded into 96-well plates at a density of 10^5^ cells/well overnight. Subsequently, the cells were treated with TNI, TNA, CTN, DTN, or TTN (1, 2, 4, and 8 *μ*g/ml) for 24 h, and the cytotoxicity was determined using MTT assay as previously reported.^43^

### Determination of nitrite and NO

RAW264.7 cells were plated in 24-well plates at a density of 5 × 10^5^/well overnight. After pretreatment with TTN (1, 2, and 4 *μ*g/ml) for 1 h, the cells were stimulated with LPS (1 *μ*g/ml) for 24 h. The nitrite levels in culture media were determined with Griess reagent in accordance with the manufacturer’s instructions.

Treated cells were collected and stained with DAF-FM diacetate (1 *μ*M) for 1 h. The fluorescence signal was determined by a FACScan flow cytometer (Becton-Dickinson, Oxford, UK) at the FITC channel.

### ELISA assay

After pretreatment with TTN (1, 2, and 4 *μ*g/ml) for 1 h, BMDMs, THP-1, and RAW264.7 cells were then incubated with LPS (1 *μ*g/ml) for 24 h, respectively. The levels of TNF-*α*, IL-1*β*, and IL-6 were determined by ELISA kits in accordance with the manufacturer’s instructions.

### Immunofluorescence

Immunofluorescence analysis of NF-*κ*B/p65 was conducted as previously described.^44^ In brief, 2 × 10^5^ cells were seeded into a 35 mm glass bottom SPL confocal dish (SPL Life Sciences, Gyeonggi-do, Korean)overnight. They were pretreated with TTN (4 *μ*g/ml) for 1 h, stimulated with LPS (1 *μ*g/ml) for another hour, and then fixed and stained with Hoechst3342 (1 *μ*M). Images were taken under a Leica TCS SP8 laser confocal microscope (Leica Microsystem, Wetzlar, Germany) using × 60 magnification with excitation and emission wavelengths at 588 and 615–690 nm, respectively.

### Transient transfection and luciferase assay

HEK 293T cells were seeded in a dish (10 cm i.d.) at a density of 10^6^ cells overnight. TLR4-HA and TLR4-Flag plasmids obtained from Addgene were co-transfected with Tubofect Transfection Reagents (Thermo Fisher Scientific) for 24 h. The cells were seeded into a dish (5 cm i.d.) at a density of 5 × 10^5^ overnight. Cells were pretreated with TTN (4 *μ*g/ml) for 1 h and then stimulated with LPS (1 *μ*g/ml) for 24 h before harvest.

RAW264.7 cells were cultured in 96-well plates overnight, and the iNOS-luc, NF-*κ*B-luc, and AP-1-luc plasmids were transiently transfected into cells to detect iNOS, NF-*κ*B, and AP-1-transcriptional activities in accordance with the manufacturer’s instructions. The pRL-TK plasmid was used as a control. After 48 h transfection, the cells were pretreated with TTN (4 *μ*g/ml) for 1 h and then stimulated by LPS (1 *μ*g/ml) for 24 h. The luciferase activities were determined using a Dual-Glo Luciferase Assay System Kit (Promega, Madison, WI, USA) in accordance with the manufacturer’s instructions.

### Immunoblotting and immunoprecipitation

Protein samples were collected from treated cells, and the protein concentrations were examined using a Pierce Biotechnology BCA Assay Kit (Pierce, Rockford, IL, USA). Subsequently, 800 *μ*g protein in IP lysis buffer was immunoprecipitated with Anti-HA Magnetic Beads or Protein A/G Magnetic Beads. Subsequent methods were performed following the manufacturer’s instructions. Equivalent amounts of each protein sample were subjected to SDS-PAGE gel electrophoresis and then transferred onto PVDF membranes. After blocking with 5% nonfat milk dissolved in Tris-buffered saline-Tween 20 buffer for 1 h, the membranes were probed with primary antibodies (1:1000) overnight at 4 °C and specific secondary antibodies (1:5000) for another hour at 25 °C. The signals of protein bands were detected with SuperSignal West Femto Maximum Sensitivity Substrate (Pierce Biotechnology, Rockford, IL, USA) and then visualized under a ChemiDoc MP Imaging System (Bio-Rad, Hercules, CA, USA).

### Quantitative real-time PCR (qRT-PCR)

Total RNA was extracted from each sample using Trizol. RNA (1 *μ*g) was employed in qRT-PCR using qPCR Master Mix. PCR amplification was performed using the incorporation of SYBR green. The oligonucleotide primers were listed as follows:

iNOS: 5′-GGCAGCCTGTGAGACCTTTG-3′ (forward) and 5′-GCATTGGAAGTGAAGCGTTTC-3′ (reverse);

TNF-*α*: 5′-TTCTGTCTACTGAACTTCGGGGTGATCGGTCC-3′ (forward) and 5′-GTATGAGATAGCAAATCGGCTGACGGTGTGGG-3′ (reverse);

IL-6: 5′-TCCAGTTGCCTTCTTGGGAC-3′ (forward) and 5′-GTGTAATTAAGCCTCCGACTTG-3′ (reverse);

COX-2: 5′-TGAGTACCGCAAACGCTTCTC-3′ (forward) and 5′-TGGACGAGGTTTTTCCACCAG-3′ (reverse);

IL-1*β*: 5′-GAAAGACGGCACACCCACCCT-3′ (forward) and 5′-GCTCTGCTTGTGAGGTGCTGATGTA-3′ (reverse); and

GAPDH: 5′-CATGACCACAGTCCATGCCATCAC-3′ (forward) and 5′-TGAGGTCCACCACCCTGTTGCTGT-3′ (reverse).

Steady-state mRNA levels of iNOS, TNF-*α*, IL-6, COX-2, IL-1*β*, and GAPDH were determined using the Takara Thermal Cycler Dice (Takara Bio Inc., Shiga, Japan).

### Animal experiments

BALB/c mice (male, 6–8-week old, 18–22 g) were obtained from the Experimental Animal Center of Soochow University. All mice were reared in plastic cages with food and water under standard conditions (SPF) and air filtration (22±2 °C, 12 h light/dark cycles). The study was in accordance with the Local Guide for the Care and Use of Laboratory Animals of Soochow University and was approved by the university’s Ethics Committee of Experimental Animal Center of Soochow University (No. IACUC2016-13).

For xylene-induced mice ear edema model,^45^ mice were given TTN (80 mg/kg, i.p.) for 2 h and then injected with 30 *μ*l xylene at the posterior and anterior surfaces of the right ear. The left ear was used as a control. One hour later, mice were killed by cervical dislocation under ether anesthesia, and two ear punches (7 mm, i.d.) were collected and weighted. Edema was calculated by the increase in weight of the right ear punch compared with the left ear. The ear tissues were collected and fixed in 10% formaldehyde for at least 24 h at room temperature. After dehydration in different concentrations of alcohol, tissues were embedded in paraffin and then sliced. H&E staining was performed, and sections were observed under a light microscope (Olympus, Glasgow, UK).

For septic shock model,^29^ sepsis was established by administration of LPS (20 mg/kg, i.p.). TTN (80 mg/kg, i.p.) was pretreated for 2 h before LPS injection. DEX (5 mg/kg, i.p.) was used as a positive control. The survival rate was monitored for 132 h. After 132 h, the remaining mice were killed by cervical dislocation under ether anesthesia.

For the AKI model,^19^ mice were administered LPS (10 mg/kg, i.p.) with or without TTN (20, 40, and 80 mg/kg, i.p.) pretreatment for 2 h. They were killed after LPS treatment for 12 h, and serum samples were collected. The cytokines (TNF-*α*, IL-6, and IL-1*β*) were examined using ELISA kits in accordance with the manufacturer’s instructions. The levels of BUN and creatinine were determined by Roche Modular P800 (Roche, Shanghai, China). Kidney tissues were collected and fixed in 10% formaldehyde, and H&E staining was performed. Sections were observed under a light microscope.

### Data analysis

All results were presented as means±S.D. For statistical analysis, the significance of the intergroup differences was analyzed by one-way ANOVA using the GraphPad Prism 6.0 software (GraphPad Software, San Diego, CA, USA). Statistically significant difference was defined as *P*<0.05.

## Publisher’s Note

Springer Nature remains neutral with regard to jurisdictional claims in published maps and institutional affiliations.
