# Supplementary material for: Total tanshinones exhibits anti-inflammatory effects through blocking TLR4 dimerization via the MyD88 pathway
Source: Cell Death Dis. 2017 Aug 17;8(8):e3004–. doi: 10.1038/cddis.2017.389 (PMC5596575; doi:10.1038/cddis.2017.389)
Supplement: Supplementary Figure S1 [file cddis2017389x1.docx]

**Total tanshinones exhibits anti-inflammatory effects through blocking TLR4 dimerization *via* the MyD88 pathway**

Hongwei Gao^1^, Xin Liu^1^, Wen Sun^1^, Naixin Kang^2^, Yanli Liu^2^, Shilin Yang^2^ , Qiong-ming Xu^2*^, Chunming Wang^1^, Xiuping Chen^1*^

*^1^State Key Laboratory of Quality Research in Chinese Medicine, Institute of Chinese Medical Sciences, University of Macau, Macau, China,* *and ^2^**College of Pharmaceutical Science, Soochow University, Suzhou 215123, China.*

Running title: Total tanshinones exerts anti-inflammatory effects

^*^Correspondence:

Dr. Qiong-ming Xu

Address: Department of Pharmacognosy, College of Pharmaceutical Science, Soochow University, Suzhou 215123, China

E-mail: xuqiongming@suda.edu.cn

Tel: +86-512-69561421 Fax: +86-512-65882089

Dr. Xiuping Chen

Address: Institute of Chinese Medical Sciences, University of Macau, Avenida da Universidade, Taipa, Macau, China

E-mail: xpchen@umac.mo

Tel: +853-88224679 Fax: +853-28841358

Supplementary figure


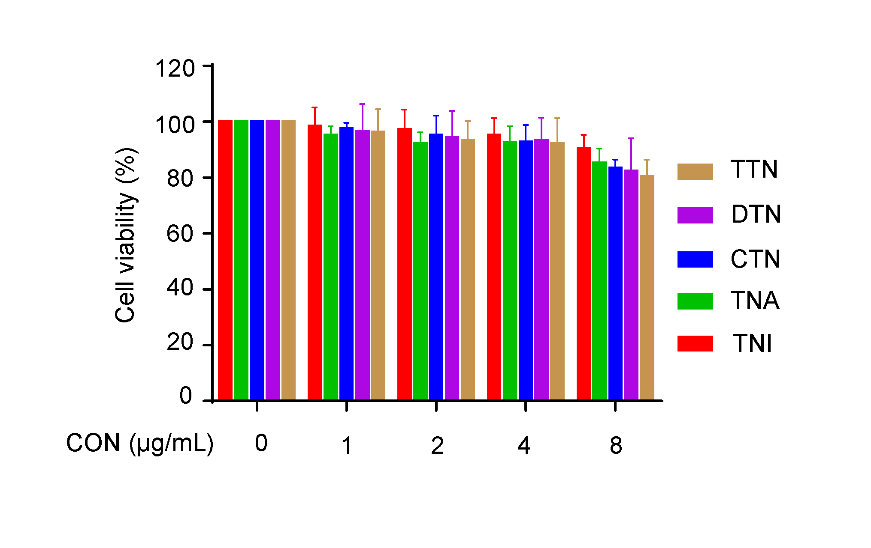


Figure S1. The cytotoxicity of TNI, TNA, CTN, DTN, or TTN in RAW264.7 cells. RAW264.7 cells were seeded into 96-well plates at a density of 10^5^ cells/well overnight. Subsequently, the cells were treated with TNI, TNA, CTN, DTN, or TTN (1, 2, 4, and 8 μg/mL) for 24 h, and the cytotoxicity was determined using MTT.
